# Rationale and design of extended cancer education for longer term survivors (EXCELS): a randomized control trial of ‘high touch’ vs. ‘high tech’ cancer survivorship self-management tools in primary care

**DOI:** 10.1186/s12885-019-5531-6

**Published:** 2019-04-11

**Authors:** Stacy N. Davis, Denalee M. O’Malley, Alicja Bator, Pamela Ohman-Strickland, Lynn Clemow, Jeanne M. Ferrante, Benjamin F. Crabtree, Suzanne M. Miller, Patricia Findley, Shawna V. Hudson

**Affiliations:** 1 Rutgers Biomedical and Health Sciences, Rutgers, the State University of New Jersey, 112 Paterson Street, Room 446, New Brunswick, NJ 08901 USA; 2 Rutgers School of Public Health, Health Behavior, Society and Policy, Piscataway, NJ USA; 3 Rutgers Cancer Institute of New Jersey, New Brunswick, NJ USA; 4 Rutgers Robert Wood Johnson Medical School, Department of Family, Medicine and Community Health, New Brunswick, NJ USA; 5 Rutgers School of Public Health, Biostatistics, Piscataway, NJ USA; 6 Rutgers School of Social Work, New Brunswick, NJ USA; 7 Fox Chase Cancer Center/Temple Health, Philadelphia, PA USA

**Keywords:** Randomized controlled trial, Electronic health (eHealth), Health coaching intervention, Cancer survivorship, Primary care, Self-management, Oncology

## Abstract

**Background:**

Breast, colorectal, and prostate cancer survivors are at increased risk for late and long-term effects post-treatment. The post-treatment phase of care is often poorly coordinated and survivors navigate follow-up care with minimal information or guidance from their healthcare team. This manuscript describes the Extended Cancer Education for Longer-term Survivors (EXCELS) in Primary Care protocol. EXCELS is a randomized controlled trial to test the efficacy of patient-level self-management educational strategies on adherence to preventative health service use and cancer survivorship follow-up guidelines.

**Methods:**

The EXCELS trial compares four conditions: (1) EXCELS-website (e.g., a mobile-optimized technology platform); (2) EXCELS-health coaching; (3) EXCELS-website and health coaching; and (4) a print booklet. Approximately 480 breast, colorectal, and prostate survivors will be recruited through the New Jersey Primary Care Research Network (NJPCRN) and New Jersey State Cancer Registry (NJSCR). Eligible survivors (diagnosed stages 1–3) must have completed active treatment, access to a phone and a computer, smartphone or tablet with internet access, and be able to speak and read English. Patient assessments occur at baseline, 6, 12, and 18 months. The primary outcomes are increased engagement in preventive health services and monitoring for cancer recurrence and treatment-related late effects.

**Discussion:**

The EXCELS trial is the first to test cancer survivorship educational self-management interventions for cancer survivors in a primary care context. Findings from this trial will inform successful implementation and engagement strategies for longer-term, post-treatment cancer survivors managed in primary care settings.

**Trial registration:**

Registered August 1, 2017 at ClinicalTrials.gov, trial # NCT03233555.

## Background

Cancer survivors, a population that has reached 15.5 million individuals in the United States, face accelerated aging due to the impact of cancer and specific treatments [1–3]. Therefore, cancer survivors need information and support to monitor their health and to engage in post-treatment care recommendations, such as an assessment of treatment late effects, surveillance for cancer recurrence, and promotion of preventive health behaviors such as vaccinations [4]. Management of post-treatment care recommendations is associated with improved health-related quality of life [3]. Several trials demonstrate that post-treatment follow-up in primary care is as effective as oncology care for clinical indicators and quality of life outcomes [5–8]. Guidelines for primary care providers offer direction for managing cancer follow-up, focusing on cancer surveillance along with late and long-term symptom management for specific cancer sites [9–11]. Approximately half of post-treatment cancer patients express additional informational needs [12]. However, the most effective modalities to present this information and empower long-term survivors to manage their health and psycho-social outcomes remain unknown [13].

Patients are increasingly using technology to communicate health issues, manage chronic disease, and search for health information [14–17]. Integrating technology into care management has the potential to enhance interactions with providers, enhance the understanding of survivorship care plans, and positively affect health outcomes [18]. Both ‘high tech’ (i.e., web-based mobile optimized technology) and ‘high touch’ (i.e., health coaching) interventions are often used to support patient self-management in chronic disease. Promising results in the areas of cervical cancer prevention and chronic disease management suggest that individuals’ interaction with technology-based interventions may impact self-management behaviors [18, 19]. Health coaching is another educational, patient-centered strategy to communicate cancer survivorship information. Health coaching models use a relational approach to promote sense-making of health information and support goal setting for health behavioral change (i.e., lifestyle, risk reduction, and self-management) [20]. The effectiveness of health coaching has been established in a variety of chronic diseases to: 1) identify patient readiness to adopt new behaviors; 2) develop patient-centered strategies for behavior change; and 3) effectively build knowledge, skills, and confidence necessary for patients to manage chronic diseases [21–25].

To date, health coaching and web-based mobile-optimized technology (or eHealth) approaches have not been tested separately or in combination to assess the impact on health maintenance and cancer-related follow-up care among cancer survivors. Thus, the **Ex**tended **C**ancer **E**ducation for **L**onger-term **S**urvivors (EXCELS) intervention—a web-based mobile optimized technology platform was developed to be used independently or with a health coaching program. The goal of EXCELS intervention is to enhance patient self-management during post-treatment survivorship among breast, colorectal, and prostate cancer survivors. This paper describes the design, methods, and data analysis plans for the ongoing randomized controlled trial (RCT) to evaluate the efficacy of EXCELS to increase engagement in preventive health services and monitoring for recurrence and late effects.

## Methods and design

### Overview and research objectives

**Fig. 1:**
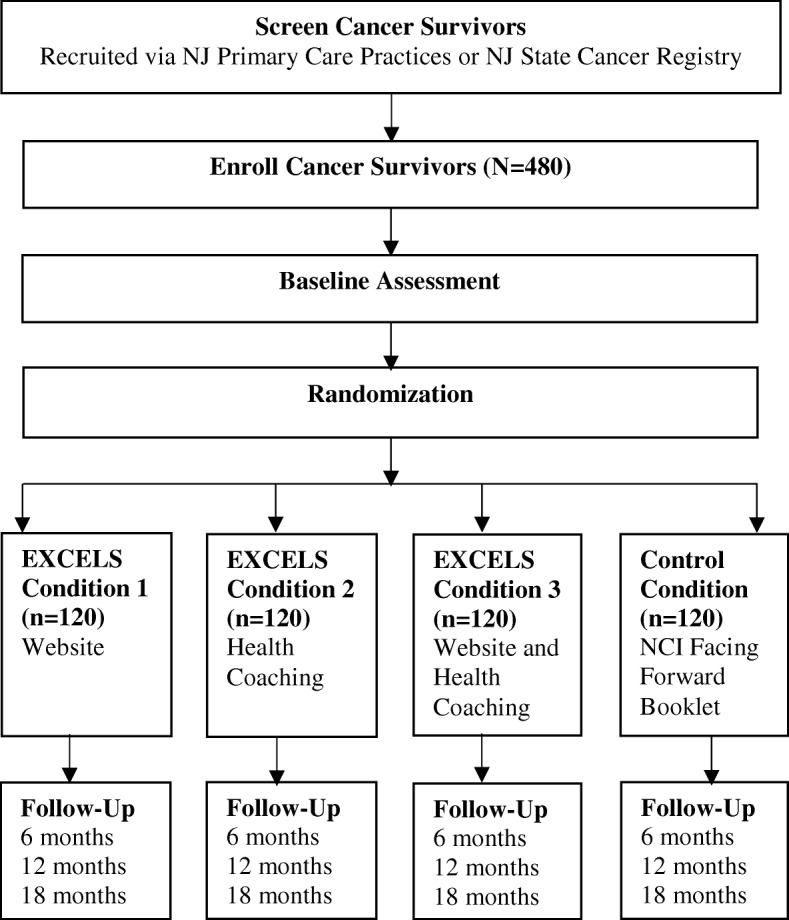
Study Schema

EXCELS is a two-phase research study to develop and evaluate the comprehensive intervention in a RCT. Phase 1 used a mixed-method, multi-step iterative process to develop intervention components. The development of the EXCELS intervention components was conducted with 45 patients and 10 clinicians. The objective of the Phase 2 is to determine the efficacy of the EXCELS intervention to increase engagement in preventive health services and monitoring for recurrence and late effects. The 18-month EXCELS trial implements a 2 × 2 factorial RCT design comprised of three intervention conditions and a comparison condition (see Fig. 1). Four hundred and eighty Breast, colorectal, and prostate cancer survivors are randomized to receive either EXCELS-website (high tech), EXCELS-health coaching (high touch), EXCELS- website and health coaching (integrated tech-touch), or a comparison condition (*National Cancer Institute Facing Forward* booklet) [26]. It is hypothesized that compared to the comparison condition, participants randomized to EXCELS-high tech, EXCELS- high touch, and EXCELS- integrated tech-touch conditions will demonstrate increased levels of engagement in preventive health services and monitoring for cancer recurrence and late effects via self-report (6 months, 12 months, and 18 months) and medical chart review for a subset of survivors (20 months). The secondary outcomes include the examination of mediators (i.e., patient activation, recurrence, and perceived intervention utility) and moderators (i.e., patient perceived cancer vulnerability and use of primary care) on the intervention’s impact via self-report.

EXCEL phase 2 ethics approval was received from the Rutgers University Institutional Review Board and the CentraState Healthcare System Institutional Review Board. Written informed consent will be obtained from all trial participants. The EXCELS trial is registered at www.clinicaltrials.gov, trial # NCT03233555*.*

### Theoretical framework

The EXCELS trial is guided by the Cognitive-Social Health Information Processing Model (C-SHIP), an integrative theoretical framework which addresses how individuals process information about cancer threats and prevention options based on their perceived vulnerability, disease and self-efficacy expectations, goal and values and coping strategies [27]. The key constructs of the C-SHIP model operationalized in EXCELS include: 1) cancer and health relevant interpretations; 2) beliefs and expectancies about cancer treatment and disease outcomes; 3) cancer and health relevant goals and values; 4) cancer and health-relevant affective and emotional states; and 5) self-regulatory competencies. EXCELS supports processing of tailored cancer survivorship information using traditional formats (e.g., print materials), new technologies, and health coaching techniques to test their impact on survivor’s adoption of cancer control and health monitoring behaviors. The objectives of the EXCELS trial are to: 1) promote accurate processing of recurrence risk and risk of secondary cancers; 2) identify new or lingering treatment-related symptoms; 3) reframe beliefs and expectations about need for risk-based preventive care for existing sequelae; 4) articulate goals to encourage consistency between patient behavior and desire; and 5) identify barriers to goal-directed decision making. To foster accurate cancer and health-related interpretations, American Cancer Society guidelines for primary care providers on breast, colorectal and prostate cancer follow-up were converted into printed health coaching manuals and web-based education materials [9–11].

### Participant recruitment and eligibility

EXCELS seeks to recruit 480 cancer survivors who are long-term survivors whom may seek follow-up care in primary care settings for management of late and long-term effects of cancer and its treatment. Two recruitment strategies are utilized to optimize patient recruitment into EXCELS, the New Jersey Primary Care Research Network (NJPCRN) and the New Jersey State Cancer Registry (NJSCR). To be eligible for EXCELS, survivors must be: 1) diagnosed with breast, colorectal, or prostate cancer (stages 1–3); 2) have completed active treatment for their cancer diagnosis (except hormonal treatment); 3) have access to a computer, a smartphone or an I-Pad/tablet equivalent with internet access; 4) have access to a phone for contact with health coach; 5) able to read and speak English; and 6) be competent to provide written informed consent. Survivors are excluded or removed from the trial if they experience a cancer recurrence or a new cancer diagnosis during their participation in the EXCELS trial.

The first recruitment strategy is within primary care practices affiliated with NJPCRN, a practice-based research network that has well established collaborative research linkages. To identify survivors, practices request a report from their billing system using 57 ICD-10 codes for breast, colorectal and prostate cancer diagnosis or history. Identified patients receive a mailed invitation from their primary care practice that also includes a letter from the study principal investigator (PI). The letter from the PI highlights trial eligibility criteria, offer three methods to ‘opt-in’ (postage-paid postcard, telephone, or email) to receive a telephone call to assess eligibility, and include the study telephone number and e-mail address. Research assistants send up to two repeat mailings after the initial mailing. Patients that meet eligibility criteria and volunteer to take part in the trial are emailed a link to complete an electronic informed consent. Participants recruited though NJPCRN will have their medical charts reviewed to determine receipt of preventive health service use and cancer related follow up care. The chart audit data will be compared to patient self-report to verify self-management behaviors. In addition, patients recruited through NJPCRN practices are asked to provide written release for cancer-related information (cancer site, stage, tumor characteristics e.g., hormonal status, Gleason score) obtained from the NJSCR. Patient cancer-related data will verify patient self-reported data at baseline and in the educational tailoring process to access potential implementation issues.

Participants are also recruited from the NJSCR, a resource established by the New Jersey Legislature to evaluate the impact of cancer at the state level (N.J.S.A. 26:2–104 et seq.). The NJSCR can only release identifiable information about individuals diagnosed with cancer for research with patient consent. Therefore, initial patient contact to determine interest and trial eligibility is through the NJSCR staff. NJSCR staff identifies patients from the registry with diagnoses of breast, colorectal and prostate cancers and contact the patient’s physician of record. The physician of record receives a mailed informational letter describing the trial and NJSCR’s intention to contact the patient for screening and trial participation. The physician of record is given a two-week window to notify the NJSCR staff of any objections to contacting the patient, (e.g., death, mental illness, severe illness, etc.) otherwise, passive physician consent is assumed. After physician notification, EXCELS recruitment packets are mailed from NJSCR to potentially eligible survivors. The contents of the packets include: 1) an invitation letter that highlights eligibility criteria for the EXCELS trial; 2) EXCELS information sheet with instructions on how to give agreement to contact (e.g., over telephone, email or by returning signed postage-paid release); 3) NJSCR frequently asked questions brochure; and 4) EXCELS trial recruitment flyer. One week after the recruitment packet is mailed a NJSCR team member calls potential participants to confirm receipt of the recruitment packet, answer questions about EXCELS, and screen for trial eligibility. Potential participants are called once per week for up to three weeks. If participants are not reached during this period, a final recruitment packet is mailed. Participants who are reached, screened, and eligible then have a consent discussion by telephone and e-mailed a link to complete an electronic informed consent. Once consented, the participant’s contact information is released to the EXCELS research team. Participants may also return a written release of information to NJSCR via postage paid envelope from the recruitment packet. In this case, participant information is securely forwarded from NJSCR to the EXCELS research team. The EXCELS research team proceeds with eligibility screening and subsequent electronic informed consent.

### Stratification and randomization

Upon completion of electronic informed consent, participants complete an electronic baseline questionnaire (see Fig. 1). After completion of baseline questionnaire, participants are randomized into one of four intervention conditions using a computer-based algorithm that will equalize groups across conditions through the DatStat software system: 1) EXCELS-high tech; 2) EXCELS- high touch; 3) EXCELS- integrated tech-touch and 4) comparison-booklet. The randomization algorithm equalizes the groups across experimental conditions, 120 participants per condition for 480 participants. The breakdown of cancer disease site is 200 breast cancer survivors, 200 prostate cancer survivors, and 80 colorectal cancer survivors.

### EXCELS intervention conditions

#### EXCELS-high tech

The EXCELS website tool tailors recommendations for follow-up care (e.g., surveillance and screening) using a user generated profile (see Table 1). The user profile is based on basic demographic information (e.g. gender, age), cancer site, cancer treatments received, time since last treatment, and smoking status. User provided information create tailored guideline concordant recommendations for follow-up cancer surveillance and screenings, preventive health tests, and exams. Participants randomized to the EXCELS-high tech condition are oriented to the website through print materials and a follow-up web orientation phone call. Within one week of baseline assessment, participants receive printed documents in the mail with instructions about how to access the website (e.g., EXCELS website tool URL), their temporary username and password, and instructions on how to change their temporary password. One week after the website access instructions are mailed, study staff conducts a web-orientation call to confirm receipt of materials and answer participant questions. During the orientation call, participants are instructed to go the EXCELS website to change their password and enter information to generate their user profile. Participants are also encouraged to watch an EXCELS website video tutorial that provides a short-orientation about the different functions of the website and demonstrates the interactivity of the different website sections. Aside from the brief web orientation call the EXCELS website tool is self-directed; participants can engage the materials as often and as much as they choose. Participants have unlimited access to the site.

**Table 1:** EXCELS Intervention “High Tech” and “High Touch” Components

EXCELS – Website Tool	EXCELS – Health Coaching
Home Page: Provide information on • General health and cancer follow-up • Managing symptoms, late and long term effects	Call 1: Introduction to EXCELS health coaching • Introduction to cancer follow-up • Survivorship Symptom Tracker • Directed learning to symptom specific health topics
Recommendations: Tailored information on • Cancer surveillance and screening • General health • Symptom awareness	Call 2: Cancer survivorship wellness planner • Cancer surveillance • Late and long-term effects • General health
To-do List: • Survivors view personalized health exams and screenings • Tracks past and future survivorship appointments	Call 3: Set wellness SMART goals • How to do it • Priority goals • Steps to achieve goals
My Healthcare Team: • Lists healthcare providers and corresponding preventive health appointments and developed questions	Call 4: Evaluate SMART goals • How to do it • Priority goals • Steps to achieve goals
Learn and Prepare: • Information related to diverse health topics • Healthy Lifestyle and coping • Develop questions to ask their providers regarding health topics, symptoms, and healthy lifestyle	

Once users generate a profile, an interactive “To-Do” list is generated with suggested dates of recommended tests and exams based on general and cancer site-specific guidelines. The purpose of the list is to encourage participants to organize, track, and schedule upcoming and past-due clinical exams and tests. Educational materials tailored by cancer site and gender are provided on key topics (e.g., general health, late and long-term effects, and lifestyle modification) in the “Learn and Prepare” section. For each key topic in this section descriptive information, links to resources, and suggested healthcare provider questions are provided to prime productive interactions with their healthcare team. The “My Healthcare Team” section allows users to list their provider information and list questions or notes they want to bring up during their next appointment. The questions suggested for each key topic in the “Learn and Prepare” section can be linked to the “My Healthcare Team,” segment of the website.

#### EXCELS-high touch

The EXCELS – high touch condition is a phone-based health coaching program that guides cancer survivors to increase their adherence to cancer survivorship (e.g., symptom management and monitoring for recurrence) and general prevention care guidelines, set self-management goals, and develop strategies to communicate their symptom management or care goals with their healthcare team (see Table 1). The health coaching focuses on activating survivors to engage and manage their health. Upon randomization into the EXCELS-high touch condition, participants are mailed an introductory packet which contains contact information for the health coach and the EXCELS health coaching workbook. Participants receive a call from their health coach to schedule their upcoming health coaching call.

Participants receive four health coaching phone calls over the course of 12 weeks from their assigned health coach. Each coaching call lasts approximately 20 min. Guided through the process by the health coach and aided by the workbook, each call has a distinct goal. The goal of the first call is to develop rapport, orient the participants to the different aspects of survivorship care (e.g., cancer screening and recurrence monitoring, coping, etc.) and gain an understanding of what post-treatment symptoms, if any the participant is managing. During the second call, the health coach guides the patient through a “survivorship wellness planner” to assess patient surveillance, follow-up needs, and identify exams or tests that may be needed. The third call focuses on the process of setting a health-related goal and identifying strategies for successful implementation toward reaching that goal. The fourth call focuses on reflecting on the barriers and facilitators to achieving health-related goals. The health coach is not a content expert; rather, the coach attends to the process of facilitating patient expression of their care needs and collaboratively planning on how patients can engage with their healthcare team.

#### EXCELS-integrated tech-touch

Participants randomized to this condition receive both the EXCELS- high tech and EXCELS- high touch condition materials (see Table 1). The EXCELS integrated website tool and health coaching program allow patients to engage in choice based informational preferences (e.g., print material in coaching manual or integrated website) and the process of engaging this material is guided by the health coach. Upon randomization, participants in the integrated tech-touch condition are mailed an introductory packet. This packet includes the health coaching manual, assignment, and web orientation materials for the EXCELS website. Participants are oriented to the website during the initiation call with the health coach where the first coaching call is scheduled. Since participants have continuous access to the EXCELS website and the health coaching manual, they may refer to this information in their preferred method. The health coaching calls follow the same structure as EXCELS-high touch; however the process of symptom management, assessment of surveillance needs, and health goals that include scheduling and having conversations with health providers can be captured in either or both the print and web-based formats. Further, health coaches can assist participants in how to use the integrated components of the website based on their goals, needs, and preferences.

#### Comparison condition

This condition is intended as an augmented standard care condition that controls for the basic informational content of the interventions. Within one week of the baseline assessment, participants randomized to the comparison condition are mailed the National Cancer Institute’s (NCI) print-based educational brochure for cancer survivors, “Facing Forward: Life after Cancer Treatment” booklet. This 61 page booklet was developed with input from cancer experts and patients and was designed for adults of different ages and types of cancers [26]. The booklet addresses the following informational domains: physical and emotional functioning, interpersonal relationships, life perspectives, and practical concerns.

### Measures

Participants complete self-administered questionnaires at baseline, 6-months, 12-months, and 18-months post-baseline. Each questionnaire is administered as an online survey through DatStat software that takes approximately 30 min to complete. Reminder emails are sent to enrolled participants one week before the scheduled follow-up assessment date. Demographic characteristics (e.g., gender, race/ethnicity, age, income, smoking status, etc.) are assessed at baseline only. Participants receive a $20 gift card for each of the four questionnaires completed. Variable selection was guided by the C-SHIP model constructs. Measure selection was guided by empirical evidence of instrument reliability, validity, and sensitivity to change. A list of primary outcomes, mediators, and moderators, along with their associated data source and time interval are provided in Table 2. These data are captured through patient self-report and a confirmatory medical chart review in the subset of participants recruited from the NJPCRN.

**Table 2:** Summary of EXCELS measures collected via self-report and medical chart review

Variable	Description	C-SHIP^a^ Components	Assessment (months)			
			Baseline	6	12	18
Primary Outcomes: Use of Preventive Health Services						
Cancer recurrence	Author adapted scales	5	X	X	X	X
Preventive Cancer Screening	author adapted scales	5	X	X	X	X
Vaccinations (Influenza, Pneumonia)	author adapted scales	5	X	X	X	X
Well -visits	author adapted scales	5	X	X	X	X
Mediators and Moderators						
Cancer Risk Perceptions	author adapted scales	2	X	X	X	X
Cancer Worry (Fear of recurrence)	Cancer Worry Scale	1–5	X	X	X	X
Demographics	author adapted scales		X			X
Family and Provider Care Responsibility	author adapted scales		X	X	X	X
Information Technology Use for Health	The eHealth Literacy Scale (eHEALS)	2	X	X	X	
Health Literacy	Chew Health Literacy		X		X	
Health-Related Quality of Life	Patient-Reported Outcomes Measurement Information System (PROMIS) Global Health Measure	1–5	X	X	X	X
Patient Activation	Patient Activation Measure (PAM)	2–5	X	X	X	X
Use of PCP and Cancer Treatment Team for Follow-up Care	Patient-Centered Medical Home (PCMH) scale	2–3	X			
Patient Satisfaction	Patient Satisfaction Questionnaire Short Form (PSQ-18)	2–3	X	X	X	
Perceived Cancer Vulnerability and concern about co-morbid disease	Follow-Up Care Use and Health Outcomes of Cancer Survivors (FOCUS)	1	X		X	
Perceived Utility of Intervention	author adapted scales	1–5		X	X	X
Technology Ownership & Use	Health Information National Trends Survey (HINTS)	2	X	X	X	
Treatment late-effects/ Co-morbidity monitoring	Charlson Comorbidity Index (CCI)		X	X	X	X
Process Evaluation						
Intervention Use and Satisfaction	Author adapted scales	5		X		

^a^C-SHIP Components: (1) cancer and health-relevant interpretations; (2) beliefs and expectations about cancer treatment and disease outcomes, as well as beliefs about one’s own self-efficacy in dealing with health related challenges; (3) cancer and health relevant goals and values; (4) cancer and health-relevant affective and emotional states; and (5) self-regulatory competencies

## Primary outcomes

### Use of preventive health services

Preventive services use is measured as adherence to evidence based guideline care based on author adapted measures [28] updated using criteria from the Physician Quality Reporting System (PQRS) measures [29]. Eligibility for guideline-related services is determined based on the medical assessment, including patient/family history, laboratory and physical findings in the medical records and patient surveys. Measures of preventive health services include influenza vaccination for patients 50 or older, pneumonia vaccination for patients 65 and older, receipt of annual physical exams, breast cancer screening with mammography, and colorectal cancer screening. Adherence is computed as a percentage of the eligible guideline-related services each participant has received.

### Monitoring for cancer recurrence and chronic conditions

Cancer recurrence is assessed via patient self-report “At any time since you were diagnosed with your primary cancer, did a doctor or other health professional tell you that your cancer had come back (did you have a recurrence)?” The validated Charlson Comorbidity Index (CCI) will assess late effects of cancer treatment and comorbid conditions [30]. The CCI, based on 19 chronic conditions, is a weighted index that takes into account the number and seriousness of comorbid diseases. We will also assess additional chronic diseases via participant self-assessment.

## Secondary outcomes

Cancer risk perceptions is assessed with two single-item measures. The first item “What do you think are the chances that your cancer will come back or get worse within the next 10 years?” is assessed on a five-point scale ranging from “very low” to “very high” [30]. The second item “Compared with the cancer risk in the general population, would you say that your risk of the second cancer is...” is assessed on a five-point scale ranging from “much lower” to “much higher” [31].

Cancer worry (fear of recurrence) is determined by a four item scale that reflect the survivor’s worries directly related to cancer: 1) “I worry about future diagnostic tests”; 2) “I worry about my cancer coming back”; 3) “I worry about another type of cancer”; and 4) “I worry about future diagnostic tests”; and 4) “I worry about another type of cancer” [32].

Demographics is collected via patient self-report of age, sex, race, ethnicity, income, level of education, health insurance, employment status, foreign born status, marital status, family history of cancer, personal cancer history, cancer treatment, height, and weight.

Information technology use for health is assessed with the *eHealth Literacy Scale* (eHEALS) [33]. The 8-item eHEALS is a validated scale that aims to reflect the individuals’ own perception of their knowledge and skills at using eHealth information and determine whether an eHealth approach is suited to the individual.

Health literacy is assessed with the validated 3-item *Chew Health Literacy Scale* (CHL) [34]. The CHL assess patient health literacy problems due to reading, understanding, and filling out forms.

Health related quality of life is measured using the *Patient Reported Outcomes Measurement Information System* (PROMIS) to collect information regarding general physical health and health related quality of life [35]. The 9-item PROMIS is a reliable and valid survey that has physical health and emotional well-being subscales.

Family and provider care responsibility is assessed with a 3-item author adapted scale [28]. This scale assesses whom among the healthcare professionals involved is considered to be in charge of patient’s care and whom among the family system (e.g. patient, spouse, partner, or child) is in charge of the patient’s care.

Patient activation is assessed with the *Patient Activation Measure* (PAM) [36]. The PAM is a 13-item validated measure that gauges patient knowledge, skills and confidence related to managing one’s own health and healthcare.

Patient-centered medical home utilization is determined by *Patient-Rated Patient-Centered Medical Home* (PCMH) Scale [37]. The 20-item PCMH assesses primary care provider and cancer specialist involvement in follow-up care.

Patient satisfaction is assessed with the *Patient Satisfaction Questionnaire III* (PSQ-18) [38]. The PSQ-18 is a 18-item validated measure to assess satisfaction with their primary care physicians and their oncology specialists.

Perceived cancer vulnerability and concerns about comorbid conditions and late effects is evaluated using measures from a series of questions from the NCI’s Office of Cancer Survivorship’s Follow-Up Care Use and Health Outcomes of Cancer Survivors (FOCUS) [39, 40].

Technology ownership and use is assessed using questions adapted from the *Health Information National Trends Survey* (HINTS) [41]. Online health information seeking behavior in the past 12 months is assessed with the question “Do you ever go online to access the Internet or World Wide Web, or to send and receive email?” Participants who respond yes will be asked a series of questions about their online information seeking behavior during the past 12 months, including whether they used the Internet to look for health or medical information for themselves or for someone else. Use of the Internet for health related behaviors and specific information seeking efforts will also be assessed, e.g. “Used email or the Internet to communicate with a doctor or doctor’s office”. To assess health information seeking, participants will be asked “The most recent time you looked for information about health or medical topics, where did you go first?”

## Process measures

We will use two measures to assess experience with and usability of the intervention. The perceived utility of the intervention will be tested with an author adapted measure that assesses the helpfulness of components of the intervention in self-management domains (e.g. managing emotional concerns, managing physical concerns, etc.). The usability of the EXCELS website tool will be further assessed using the *System Usability Scale* (SUS), a validated, industry standard scale used to test a variety of products and services, including websites and mobile phones [42].

### Sample size (power analysis)

The statistical power calculation of the trial is based on the primary outcome of increased engagement in preventive health services and monitoring for recurrence and late effects. The key assumptions for the power calculations stem from the literature and preliminary data. Given the trial’s hypothesis participants randomized to EXCELS intervention conditions will demonstrate greater effects over those in the comparison condition. Assuming a Pearson chi-square test for comparing rates of adherence to recommendations between two arms, we calculated the power attained using 120 individuals per condition arm. EXCELS has four condition arms, thus six pairwise comparisons of conditions and each comparison can be tested at the 0.00833 level (Bonferroni correction 0.05/6) to attain a 0.05 family-wise error rate. We conservatively assume that across conditions, the rate of adherence is 50%. With a rate ratio of 2.0 (0.66 versus 0.33), the power to detect an effect of treatment is 85.9%. Power would be greater if rates of adherence for the comparison group are less than 0.33 or at 0.50 or greater.

### Statistical analysis

The primary outcomes are preventive health services engagement, monitoring for recurrence and late effects. Descriptive and exploratory analyses will be conducted for all baseline measures to characterize the sample and stratify by treatment arm to describe the inferential population. Primary analyses will be conducted with analysis of variance (ANOVA) to compare the proportions of preventive and surveillance services used and proportions of potential late effects tracked for which the participant was eligible, between participants in intervention conditions and the comparison condition. To examine mediators of primary outcomes, ANOVA models will be expanded to include a main effect of the mediator as well as interaction between the conditions and the potential moderator. F-tests of the interaction will assess any moderating effects. If significant, linear combinations of regression coefficients will estimate the effect of the intervention at varying levels of the moderator. For each potential mediator, the four component steps of the mediation model will be examined via regression models. Bootstrap procedures will formally test for significance and estimate the percent contribution of the mediating pathway to the main effect of the intervention. All participants will be informed by e-mail about the final results of the trial. Scientific publications will also be planned.

### Data management

Participants using a secure website will complete online surveys using a secure website (hosted on DatStat servers). The software allows for research study personnel to be assigned data access and privileges specific to their role on the study. Databases for participant recruitment, tracking, medical records review data, and survey data are maintained by the Rutgers Cancer Institute of New Jersey, Population Science Research Support Core using HIPAA-compliant DatStat software.

### Data and safety monitoring

Rutgers University maintains specific formal policies and infrastructure that have been developed to assist investigators with ensuring that research participants and research data are adequately protected from harm. In accordance with these policies, for this study we will use standard procedures and infrastructure for data and safety monitoring. We will not use a protocol-specific Data Safety Monitoring Board since the study involves minimal risk. Thus, the oversight committees is adequate to ensure participant safety and data integrity. Study protocol will be submitted for annual review to the Rutgers University Institutional Review Board.

## Discussion

Comprehensive self-management in the primary care setting post-treatment requires the provision of care plans along with empowerment of cancer survivors to manage their health and complex post-treatment care recommendations [43]. The EXCELS trial is designed to empower breast, colorectal, and prostate cancer survivors to enhance their engagement in preventive services and adherence to post-treatment monitoring. The EXCELS trial design is based on the synergistic use multi-media and health coaching to encourage survivors to engage in choice based informational preferences, provide prompts to assist their communication with diverse healthcare providers, and provide an opportunity to manage their follow-up recommendations and track upcoming clinical appointments.

Of note, EXCELS is one of a relatively few cancer-focused and general preventive educational intervention studies designed for survivors who have transitioned out of oncology care and into primary care settings for post-treatment follow-up [44]. To our knowledge, existing cancer survivor interventions do not incorporate an integrated website tool and health coaching program to address ongoing cancer surveillance along with late and long-term symptom management and addressing psychosocial needs. The EXCELS trial is the first to integrate and measure the social interactional aspects of adaption to health challenges that are most significant for intervention effectiveness on patient-reported outcomes. EXCELS facilitates social interactions by: 1) providing accurate survivorship information; 2) setting realistic post-treatment expectations; 3) promoting self-efficacy through providing information and training to maximize self-management knowledge and skills; 4) exploring patient’s health goals and encouraging behavior consistent with the health goals; and 5) promoting emotional support.

Novel strategies are needed to provide information and support to survivors to mitigate long-term risks [45]. The EXCELS intervention prepares survivors for future encounters with their healthcare team by providing survivors with an opportunity to proactively and systematically manage their post-treatment preventive health services and cancer surveillance. Findings from the EXCELS trial will offer insights on how to best engage, offer information and support to cancer survivors, particularly those survivors who have progressed into longer-term cancer survivorship. Successful completion of the EXCELS trial will provide evidence for intervention efficacy which can be disseminated in diverse survivors in multiple settings. The strategy of coupling a “high touch” health coaching intervention and a “high tech” mobile web internet based intervention has potential to yield an effective, highly disseminable intervention that has long-term potential to benefit a large number of survivors. The results of this intervention should inform the design of programs to enhance patient activation and engagement in their follow-up care. Future plans include exploring the generalizability of the EXCELS intervention in additional locations, languages, and survivors. This trial fills a growing unmet need to enhance extended follow-up care for cancer survivors following active treatment. Thus, improving cancer surveillance and chronic disease prevention and management.

## Abbreviations

ANOVA: analysis of variance
C-SHIP: Cognitive-social health information processing model
EXCELS: Extended cancer education for longer-term survivors
mHealth: mobile health
NJPCRN: New Jersey primary Care research network
NJSCR: New Jersey State cancer registry
PI: Principal investigator
